# Antibacterial activities and antioxidant capacity of *Aloe vera*

**DOI:** 10.1186/2191-2858-3-5

**Published:** 2013-07-19

**Authors:** Fatemeh Nejatzadeh-Barandozi

**Affiliations:** 1 Department of Horticulture, Faculty of Agriculture, Khoy Branch, Islamic Azad University, P.O. Box 58168–44799, Khoy, Iran

**Keywords:** *Aloe vera*, Antibacterial activities, Antioxidant capacity, Gas chromatography, Mass spectrometry

## Abstract

**Background:**

The aim of this study was to identify, quantify, and compare the phytochemical contents, antioxidant capacities, and antibacterial activities of *Aloe vera* lyophilized leaf gel (LGE) and 95% ethanol leaf gel extracts (ELGE) using GC-MS and spectrophotometric methods.

**Results:**

Analytically, 95% ethanol is less effective than ethyl acetate/diethyl ether or hexane (in the case of fatty acids) extractions in separating phytochemicals for characterization purposes. However, although fewer compounds are extracted in the ELGE, they are approximately 345 times more concentrated as compared to the LGE, hence justifying ELGE use in biological efficacy studies *in vivo*. Individual phytochemicals identified included various phenolic acids/polyphenols, phytosterols, fatty acids, indoles, alkanes, pyrimidines, alkaloids, organic acids, aldehydes, dicarboxylic acids, ketones, and alcohols. Due to the presence of the antioxidant polyphenols, indoles, and alkaloids, the *A. vera* leaf gel shows antioxidant capacity as confirmed by ORAC and FRAP analyses. Both analytical methods used show the non-flavonoid polyphenols to contribute to the majority of the total polyphenol content. Three different solvents such as aqueous, ethanol, and acetone were used to extract the bioactive compounds from the leaves of *A. vera* to screen the antibacterial activity selected human clinical pathogens by agar diffusion method. The maximum antibacterial activities were observed in acetone extracts (12 ± 0.45, 20 ± 0.35, 20 ± 0.57, and 15 ± 0.38 nm) other than aqueous and ethanol extracts.

**Conclusion:**

Due to its phytochemical composition, *A. vera* leaf gel may show promise in alleviating symptoms associated with/or prevention of cardiovascular diseases, cancer, neurodegeneration, and diabetes.

## Background

*Aloe vera* (L.) Burm.f. (*Aloe barbadensis* Miller) is a perennial succulent xerophyte, which develops water storage tissue in the leaves to survive in dry areas of low or erratic rainfall. The innermost part of the leaf is a clear, soft, moist, and slippery tissue that consists of large thin-walled parenchyma cells in which water is held in the form of a viscous mucilage [1]. Therefore, the thick fleshy leaves of aloe plants contain not only cell wall carbohydrates such as cellulose and hemicellulose but also storage carbohydrates such as acetylated mannans [2].

*A. vera* has been used for many centuries for its curative and therapeutic properties, and although over 75 active ingredients from the inner gel have been identified, therapeutic effects have not been correlated well with each individual component [3]. Many of the medicinal effects of aloe leaf extracts have been attributed to the polysaccharides found in the inner leaf parenchymatous tissue [4,5], but it is believed that these biological activities should be assigned to a synergistic action of the compounds contained therein rather than a single chemical substance [6]. *A. vera* is the most commercialized aloe species, and processing of the leaf pulp has become a large worldwide industry. In the food industry, it has been used as a source of functional foods and as an ingredient in other food products, for the production of gel-containing health drinks and beverages. In the cosmetic and toiletry industry, it has been used as base material for the production of creams, lotions, soaps, shampoos, facial cleansers, and other products. In the pharmaceutical industry, it has been used for the manufacture of topical products such as ointments and gel preparations, as well as in the production of tablets and capsules [7,8]. Important pharmaceutical properties that have recently been discovered for both the *A. vera* gel and whole leaf extract include the ability to improve the bioavailability of co-administered vitamins in human subjects [9]. Due to its absorption enhancing effects, *A. vera* gel may be employed to effectively deliver poorly absorbable drugs through the oral route of drug administration. Furthermore, the dried powder obtained from *A. vera* gel was successfully used to manufacture directly compressible matrix-type tablets. These matrix-type tablets slowly released a model compound over an extended period of time and thereby showing potential to be used as an excipient in modified release dosage forms [10].

Apart from *Aloe* being used extensively in the cosmetic industry, it has been described for centuries for its laxative, anti-inflammatory, immunostimulant, antiseptic [11], wound and burn healing [12], antiulcer [13], antitumor [14], and antidiabetic [15] activities. These treatments are based on anecdotal evidence or research findings done almost exclusively on *A. vera.* Different *Aloe* species would have various phytochemical contents, health benefits, and possible toxicities. Hence, it is of relevance for scientists, industry, and rural communities not only to research the relevant medicinal uses of their indigenous *Aloe* species but also to determine the active components and their individual or combined mechanisms of biological function. The use of 95% ethanol extracts of various *Aloe* species is extensively described in the literature for determining biological activity in the treatment and prevention of a variety of health conditions [16,17], in particular, diabetes [18,19]. In this study, we determined and compared the phytochemical contents and antioxidant capacities, antibacterial activities of *A. vera* lyophilized leaf gel and 95% ethanol leaf gel extracts using gas chromatography–mass spectrometry (GC-MS) and spectrophotometric methods of analysis. This was done not only to describe *A. vera* leaf gel extracts with regard to phytochemical contents and possible health benefits but also to compare various extraction methods for both analytical efficacy and possible biological relevance.

## Methods

### Samples

Whole, freshly cut, *A. vera* leaves (100 kg) were harvested in the month of September from farms in the National Institute of Genetic Engineering and Biotechnology (NIGEB), Tehran of Iran. The inner leaf gel was removed, homogenized, freeze-dried, and stored at −20°C until analysis. This was termed the leaf gel extract (LGE) for the purpose of this study. Approximately half of the LGE was used for the preparation of a 95% ethanol extract as described previously [19]. This was termed the 95% ethanol leaf gel extract (ELGE).

## Material

All analytical standards were purchased from Sigma-Aldrich (St. Louis, MO, USA), and phenol reagent and other reagent chemicals and all of the organic solvents used were of ultrahigh purity which were purchased from Merck (Darmstadt, Germany).

### Ethyl acetate/diethyl ether extraction

The internal standard, 3-phenylbutyric acid (25 mg/50 mL), was added to 25 mg of finely ground LGE and ELGE, followed by the addition of 1 mL of sodium acetate buffer (0.125 M). β-Glucuronidase (30 μL) was added, and the sample was vortexed and incubated overnight at 37°C. The sample was extracted with 6 mL of ethyl acetate followed by 3 mL of diethyl ether. The organic phase was collected after each extraction via centrifugation. The organic phase from each extraction was pooled and dried under nitrogen. The dried extract was derivatized with bis(trimethylsilyl)trifluoroacetamide (BSTFA, 100 μL), trimethylchlorosilane (TMCS, 20 μL), and pyridine (20 μL) at 70°C for 30 min. After cooling, 0.1 μL of the extract was injected into the GC-MS via splitless injection.

### Fatty acid extraction

Heptadecanoic acid (72 mM), as an internal standard, was added to 25 mg of LGE and ELGE followed by 100 μL of a 45-mM solution of butylated hydroxytoluene and 2 mL of methanolic HCl (3 N). The samples were then vortexed and incubated for 4 h at 90°C. After cooling to room temperature, the sample was extracted twice with 2 mL of hexane, dried under a nitrogen stream, and finally resuspended with 100 μL of hexane, 1 μL of which was injected onto the GC-MS via splitless injection.

### Gas chromatography–mass spectrometry

An Agilent 6890 GC ported to a 5973 mass selective detector (Santa Clara, CA, USA) was used for the identification and quantification of individual fatty acids. For the acquisition of an electron ionization mass spectrum, an ion source temperature of 200°C and electron energy of 70 eV were used. The gas chromatograph was equipped with an SE-30 capillary column (Agilent), a split/splitless injection piece (250°C), and direct GC-MS coupling (260°C). Helium (1 mL/min) was used as the carrier gas. The oven temperature program for analyzing the ethyl acetate/diethyl ether extract was an initial oven temperature of 40°C and was maintained for 2 min, followed by a steady climb to 350°C at a rate of 5°C/min. For the fatty acid analysis, an initial oven temperature of 50°C was maintained for 1.5 min and then allowed to increase to 190°C at a rate of 30°C/min. The oven temperature was maintained at 190°C for 5 min and then allowed to increase to 220°C at a rate of 8°C/min. The oven temperature was again maintained for 2 min and finally ramped to 230°C at a rate of 3°C/min and maintained for 24 min at this temperature.

### Total polyphenol assay

The total polyphenol content of the extracts were determined according to the Folin-Ciocalteu procedure [20]. Briefly, 10 mg of finely ground LGE or ELGE was dissolved in 200 μL of H2O in a test tube followed by 1 mL of Folin-Ciocalteu's reagent. This was allowed to stand for 8 min at room temperature. Next, 0.8 mL of sodium carbonate (7.5%, *w*/*v*) was added, mixed, and allowed to stand for 30 min. Absorption was measured at 765 nm (Shimadzu UV-1601 spectrophotometer, Kyoto, Japan). The mean total phenolic content (*n* = 3) was expressed as milligrams of gallic acid (Sigma-Aldrich) equivalents per 100 g of wet and dry mass (mg of GAE/100 g) (standard deviation (SD)).

### Total flavonoid assay

The total flavonoid content was measured using the AlCl3 colorimetric assay [21] with some modifications. Briefly, 10 mg of LGE or ELGE was dissolved in 1 mL of H2O, to which 60 μL of 5% (*w*/*v*) NaNO2 was added. After 5 min, 60 μL of a 10% (*w*/*v*) AlCl3 was added. In the sixth minute, 400 μL of 1 M NaOH was added, and the total volume was made up to 2 mL with H_2_O. The solution was mixed well, and the absorbance was measured at 510 nm against a reagent blank. Concentrations were determined using a catechin (Sigma-Aldrich) solution standard curve. The mean total flavonoid content (*n* = 3) was expressed as milligrams of catechin equivalents (CE) per 100 g of wet and dry mass (mg of CE/100 g) (SD).

### Oxygen radical absorbance capacity

Oxygen radical absorbance capacity (ORAC) analyses of hydrophilic and lipophilic compounds in LGE and ELGE were performed as described previously [22]. The analysis of lipophilic compounds was aided by the addition of randomly methylated β-cyclodextrin as a solubility enhancer as described before [23]. Briefly, in a volume of 200 μL, the reaction contained 56-nM fluorescein (Sigma-Aldrich) as a target for free radical attack by 240-nM 2,2′-azobis(2-amidinopropane)dihydrochloride (Sigma-Aldrich). A BioTEK fluorescence plate reader (FL-600, Winooski, VT, USA) was used, and the decay of fluorescence of fluorescein (excitation, 485 nm; emission, 520 nm) was measured every 5 min for 2 h at 37°C. Costar black opaque (96 well) plates (Thermo Fisher Scientific, Waltham, MA, USA) were used in the assays. Trolox (Sigma-Aldrich) was used as standard at a range between 0 and 20 *í*M with a polynomial (second order) curve fit analysis. Mean values (*n*) 3) of antioxidant capacities were expressed as micromoles of Trolox equivalents (TE) per gram of wet and dry mass (SD).

### Ferric reducing antioxidant power

Ferric reducing antioxidant power (FRAP) values were determined essentially as described previously [24]. Briefly, the reduction of a Fe^3+^-2,3,5-triphenyltetrazolium (Sigma-Aldrich) complex in the assay by the antioxidants in the samples was monitored at 593 nm. As a standard, FeSO4 (Sigma-Aldrich) was used, and the FRAP activities of the samples were expressed as the mean (*n* = 3) micromoles of Fe^2+^ per gram of wet and dry mass (SD).

### Antibacterial activity of *Aloe vera*

The antibacterial studies were carried out by disc diffusion technique [20]. The sterile nutrient agar plates and potato dextrose agar plates were prepared. The bacterial test organisms like *Staphylococcus aureus*, *Streptococcus pyogenes*, *Pseudomonas aeruginosa*, and *Escherichia coli* were spread over the nutrient agar plates using separate sterile cotton buds. After the microbial lawn preparation, three different extracts (20 grams of powdered plant materials mixed with 100 ml of various solvents (distilled water, ethanol, and acetone solution)) of plant disc were placed on the organism-inoculated plates with equal distance; control discs were also prepared. All bacterial plates were incubated at 27°C for 24 h. The diameter of the minimum zone of inhibition was measured in millimeter. For each test, three replicates were performed.

## Results and discussion

The compounds identified and their quantities in the *A. vera* LGE and ELGE are summarized in Table 1. Of all the compounds identified, the groups of compounds best described for their health benefits are the phenolic acids/polyphenols, sterols, fatty acids, and indoles. Apart from these, various alkanes, pyrimidines, alkaloids, organic acids, aldehydes, dicarboxylic acids, ketones, and alcohols were also identified. Although the extraction methods used in this study were not selected to target alcohols, a few of these were also identified. One would, however, expect a far larger variety of alcohols to occur in *Aloe* and in far higher concentrations. For better extraction of these, headspace isolation by simultaneous purging should be used as described previously [25]. However, by employing this method, one would extract far less of the other biologically important health-associated compounds. Therefore, to accomplish the aims of our study, alternative extraction procedures were used as described under the ‘Methods’ section using ethyl acetate/diethyl ether and hexane.

**Table 1:** Concentrations of GC-MS identified compounds from LGE and 95% ELGE

	**Concentration (ppm)**		
**Compound**	**LGE (per dry mass LGE)**	**ELGE (per dry mass LGE**	**ELGE (per dry mass ELGE)**
Phenolic acids/polyphenols			
Phenol	14.32	30.12	1.3 × 10^4^
Vanillic	58.60	24.34	8.5 × 10^3^
Homovanillic	18.55	13.26	5.0 × 10^3^
Protocatechuic	163.21	42.33	1.6 × 10^4^
3,4-Dihydroxyphenylacetic	7.54		
5-Methoxyprotocatechuic	2.5		
Syringic	25.54		
Sinapic	32.68		
*p*-Coumaric	450.87		
Isoferulic	52.90		
Ferulic	88.67	4.2	1.5 × 10^3^
Aloe emodin	87.79		
4-Phenyllactic	11.02		
4-Ethylphenol	10.12	32.21	1.2 × 10^4^
Hydrocinnamic	36.50		
*p*-Salicylic	186	59.2	1.8 × 10^4^
Benzoic	870.1	5,507	1.9 × 10^6^
Phenylpyruvic		6.50	2.3 × 10^5^
Hydro-p-coumaric	15.31		
Alcohols			
2-Butanol	13.65		
Glycerol	340.9		
Phenylethanol	86.56		
Aldehydes			
Benzaldehyde	56.34	72.5	2.5 × 10^4^
*m*-Tolualdehyde	18.21		
Organic acids			
Lactic	148	202.1	7.1 × 10^4^
Glycolic	93.1		
Pyruvic		88.1	3.1 × 10^4^
Furoic	57.43		
Phosphoric		341.2	1.2 × 10^5^
Succinic	383	117.6	4.1 × 10^4^
2-Methylsuccinic	62.1		
Picolinic		281	9.7 × 10^4^
Malic	46.7		
Tartaric	18.3		6.3 × 10^3^
Isonicotinic	40.21		
2-Hydroxybutyric		2.1	829.92
Alkanes			
1,3-Dihydroxybutane	10.22	10.56	3.7 × 10^3^
Pyrimidines			
Uracil	697.23		
Thymine	429.76	189.11	6.3 × 10^4^
Fatty acids			
Lauric (C12:0)	0.32		
Myristic (C14:0)	0.74		
Palmitoleic (C16:1)	1.32	0.19	65.70
Linoleic (C18:2 n-6)	102	0.42	143
Indoles			
Indole-3-acetic acid	2.80		
Alkaloids			
Hypoxanthine	27.65		
Ketones			
Acetophenone	8.02		
Sterols			
Cholestanol	24.32	12.99	4.6 × 10^3^
β-Sitosterol	1,604.5		
Dicarboxylic acids			
Azelaic	0.02		
Undecanedioic	0.04		

A general comparison of the phytochemical contents of the LGE and ELGE, calculated per LGE dry mass, shows that with the exception of a few compounds, far fewer compounds and at lower concentrations are extracted from 95% ethanol extracts than directly from the LGE using ethyl acetate/diethyl ether or hexane. The occurrence of higher concentrations of a few compounds from the ELGE is most probably due to matrix protein conformation changes and precipitation by the ethanol, hence making extraction of these protein-associated compounds easier [26]. However, when the concentrations are quantified for the individual compounds occurring in the ELGE per dry mass of ELGE, the concentrations for the compounds extracted are approximately 345 times higher than those for the same compounds occurring in the lyophilized LGE. Similarly, higher concentrations of total polyphenols, total flavonoids, and total non-flavonoids, as well as higher antioxidant capacities using ORAC and FRAP analyses (Table 2) are seen in the ELGE extracts. Additionally, these values are again far less when quantified per LGE dry mass. This indicates that from an analytical perspective, 95% ethanol is in general less effective than direct ethyl acetate/diethyl ether or hexane extractions (in the case of fatty acids) for the phytochemical characterization of *Aloe* species. However, the results also indicate the ELGE allows for effective concentration of a number of biologically active ingredients from LGE, confirming its popularity for use for testing biological activity for certain components *in vivo* and *in vitro*. Additionally, polyphenols are generally classified into flavonoids and non-flavonoids [27]. In Table 1, GC-MS analyses indicate the majority of the polyphenol compounds identified in the *A. vera* leaf gel belonging to the non-flavonoid group of polyphenols. This was confirmed by the spectrophotometic analysis of polyphenols summarized in Table 2, indicating the non-flavonoid components to contribute to 93% of the total polyphenols in the LGE and 92% in the ELGE.

**Table 2:** Concentrations of total polyphenols, flavonoids, and non-flavonoids as well as antioxidant capacity via ORAC and FRAP analyses

**Compound**	**LGE (dry mass)**	**LGE (wet mass)**	**ELGE (expressed as dry mass ELGE)**	**ELGE (expressed as dry mass LGE)**	**ELGE (expressed as wet mass LGE)**
Total polyphenols (mg of GAE/100 g ± SD)	78.2 ± 4.03	2.70 ± 0.14	413 ± 9.88	26.8 ± 0.63	0.93 ± 0.02
Total flavonoids (mg of CE/100 g ± SD)	5.3 ± 0.38	0.19 ± 0.01	33.6 ± 1.98	2.15 ± 0.13	0.08 ± 0.003
Total non-flavonoids (by calculation)	73.7 ± 0.43	2.55 ± 0.22	378 ± 6.78	24.5 ± 1.5	0.86 ± 0.02
ORAC, hydrophilic (*í*mol of TE/g)	53 ± 1.1	1.81 ± 0.04	136 ± 2.3	8.83 ± 0.16	0.30 ± 0.006
ORAC, lipophilic (*í*mol of TE/g)	ND	ND	ND	ND	ND
ORAC, total (*í*mol of TE/g)	53 ± 1.1	1.81 ± 0.04	136 ± 2.3	8.83 ± 0.16	0.30 ± 0.006
FRAP (*í*mol/g)	4.9 ± 0.25	0.17 ± 0.07	19.0 ± 0.3	1.21 ± 0.02	0.05 ± 0.001

Concentrations of total polyphenols, flavonoids, and non-flavonoids as well as antioxidant capacity via oxygen radical absorbance capacity (ORAC) and ferric reducing antioxidant power (FRAP) analyses in lyophilized aloe ferox leaf gel (LGE) and 95% ethanol leaf gel extracts (ELGE). ND, not detected.

Over the past 10 years, there has been a growing interest in the value of polyphenols among researchers and food manufacturers. This is mainly because of their antioxidant properties, their abundance in our diet, and their role in the prevention of various diseases associated with oxidative stress such as cancer, cardiovascular disease, neurodegeneration [28], and diabetes [29]. Polyphenols constitute a large class of molecules containing a number of phenolic hydroxyl groups attached to ring structures allowing for their antioxidant activities. These compounds are multifunctional and can act as reducing agents, hydrogen-donating antioxidants, and singlet oxygen quenchers [27]. All of the individual *A. vera* leaf gel antioxidant polyphenols identified in Table 1 may contribute to the prevention of the above-mentioned diseases to a greater or lesser extent. The individual contributions of these to disease prevention would, however, depend on their concentrations, antioxidant capacities, bioavailabilities, and specific mechanisms of action. Although the individual phenolic acids/polyphenols occurring in the highest concentrations were benzoic acid, *p*-toluic acid, *p*-coumaric acid, *p*-salicylic acid, protocatechuic acid, hydroxyphenylacetic acid, ferulic acid, aloe emodin, and vanillic acid, it is well-known that the protective health benefits of polyphenols are mainly through a combination of additive and/or synergistic effects between the individual compounds [30]. Consequently, those polyphenol/phenolic compounds identified in lower concentrations may also be of value. Due to the fact that the majority of the phenolic acids/polyphenols identified in *A. vera* leaf gel in Table 1 are antioxidants [27] and these compounds as a group occur in the highest concentrations, one would expect these to contribute to the majority of the antioxidant capacity measured in these extracts (Table 2). However, apart from these polyphenols, the indoles [31] and alkaloids identified [32] are also known to possess antioxidant activities and may consequently also contribute to the ORAC and FRAP values of these extracts. When interpreting the data of this nature, one should keep in mind that using the concentrations of these antioxidant compounds alone is insufficient criteria for making predictions of individual contributions to oxidative stress. As previously described, this is due to the fact that the concentrations of individual polyphenol antioxidants are not the only factor influencing antioxidant capacity; the structural arrangements (number and position of hydroxyl groups, double bonds, and aromatic rings) of these compounds also play a role [27]. Additionally, their individual contributions to ORAC and FRAP may also differ. Due to the FRAP analysis being an indication of the ferric ion reducing power of a compound or mixture and the ORAC analysis indicating the ability of a compound or mixture to scavenge free radicals, the various individual polyphenol components of the mixture may have stronger free radical scavenging abilities than reducing power, or vice versa, dependent on their chemical structures [33]. Phytosterols are another group of compounds that are well-known for their health benefits. Of the four phytosterols identified in Table 1, β-sitosterol occurred in by far the highest concentrations in the LGE, contributing to 93% of the total phytosterols identified.

### Antibacterial activity

Antibacterial activity of *A. vera* was analyzed against *S. aureus, S. pyogenes, P. aeruginosa and E. coli.* The maximum antibacterial activities were observed in acetone extract (12 ± 0.45, 20 ± 0.35, 20 ± 0.57, 15 ± 0.38) other than aqueous extract (0.00, 9 ± 0.54, 0.00, 0.00) and ethanol extract (7 ± 0.38, 20 ± 0.36, 15 ± 0.53, 0.00). Among the three bacterial organisms, maximum growth suppression was observed in *S. pyogenes* (20 ± 0.35) and *P. aeroginosa* (20 ± 0.57) when compared with *S. aureus* (12 ± 0.45) and *E. coli* (15 ± 0.38). Results are presented in Table 3. *A. vera* leaf gel can inhibit the growth of the two gram-positive bacteria *Shigella flexneri* and *Streptococcus progenies*[2]. Specific plant compounds such as anthraquinones and dihhydroxyanthraquinones as well as saponins [32] have been proposed to have direct antimicrobial activity.

**Table 3:** **Antibacterial activity of *Aloe vera***

	**Zone of inhibition (mm in diameter; mean ± SD; *n *= 3)**				
**Sample number**	**Extract**	***Staphylococcus aureus***	***Streptococcus pyogens***	***Pseudomonas aeruginosa***	***Escherichia coli***
1	Aqueous	-	9 ± 0.53	-	-
2	Ethanol	7 ± 0.37	19 ± 0.36	14 ± 0.53	-
3	Acetone	12 ± 0.45	20 ± 0.35	19 ± 0.57	14 ± 0.38

The ELGE was once again less effective in extracting these compounds, and only cholestanol was identified. However, the levels normalized to dry mass ELGE were not insignificant. Phytosterols are best described for their total cholesterol and low-density lipid cholesterol (LDL-C) lowering effects, consequently associated with reducing the risk for cardiovascular disease [26]. As summarized by Devaraj and Jialal [31] evidence for this has been observed in hypercholesterolemic, diabetic, and healthy volunteers. The mechanism proposed by which phytosterols accomplish this is by lowering cholesterol absorption due to the structural similarities these compounds share with cholesterol [27,29]. Apart from lowering cardiovascular risk factors associated with diabetes, phytosterols (*â*-sitosterol in particular) have been shown to positively affect diabetes by directly lowering fasting blood glucose levels by cortisol inhibition [30]. Additionally, phytosterols have been shown to reduce biomarkers for oxidative stress and inflammation [31], as well as to reduce cancer development by enabling antitumor responses by increasing immune recognition of cancer, influencing hormonal-dependent growth of endocrine tumors, and altering sterol biosynthesis due to the structural similarities of the phytosterols with these compounds and their substrates [32]. Phytosterols have also been shown to directly inhibit tumor growth by slowing cell cycle progression, by induction of apoptosis, and by the inhibition of tumor metastasis [32].

Long-chain polyunsaturated fatty acids (PUFAs) also have important biological functions noted to modulate risks of chronic degenerative and inflammatory diseases, of which the essential PUFAs, linolenic (C18:3 n-3) and linoleic (C18:2 n-6) acids, are best described [30,33]. Both of these were present in the *A. vera* leaf gel extracts, with linoleic acid being the major fatty acid present. However, despite this, the concentrations of these are still very low in comparison to the other compounds identified with possible health benefits and were not even detectable in the lipophilic ORAC analysis. These fatty acids may probably be too low for the *A. vera* leaf gel to contribute to health through its fatty acid composition. In conclusion, the results of this study show that from an analytical perspective, 95% ethanol is a less efficient solvent for the extraction of the phytochemical components of *A. vera* leaf gel for descriptive purposes as compared to ethyl acetate/diethyl ether or hexane (in the case of fatty acids). Although the 95% ethanol extracts contain a smaller variety of extracted compounds, their concentrations are, however, approximately 345 times higher than those of the lyophilized *A. vera* leaf gel when quantified as dry mass ELGE extract. This justifies the popularity of the ELGE for applications testing biological efficacy *in vivo* and *in vitro*. For the purpose of determining possible biological application, *A. vera* leaf gel was characterized.

## Conclusion

Various phenolic acids/polyphenols, phytosterols, fatty acids, indoles, alkanes, pyrimidines, alkaloids, organic acids, aldehydes, dicarboxylic acids, ketones, and alcohols were identified and quantified. Due to the presence of the antioxidant polyphenols, indoles, and alkaloids, the *A. vera* leaf gel shows antioxidant capacity as confirmed by ORAC and FRAP analyses. Both GC-MS and spectrophotometric analyses show the non-flavonoid polyphenols to contribute to the majority of the total polyphenol content. Due to the occurrence of the polyphenols, phytosterols, and perhaps the indoles present, *A. vera* leaf gel may show promise in alleviating or preventing the symptoms associated with cardiovascular diseases, cancer, neurodegeneration, and diabetes. This may be due to the well-documented lowering effects of these compounds on total cholesterol, LDL-C, and fasting blood glucose. These results support the current use of *A. vera* by both industry and traditional healers for the treatment of the above-mentioned diseases. However, further clinical trials regarding these claims are necessary before accurate conclusions regarding these heath benefits can be made.

## Competing interests

The author declare that she has no competing interests.
